# Supplementary figures and images for: The Use of a Chimeric Rhodopsin Vector for the Detection of New Proteorhodopsins Based on Color
Source: Front Microbiol. 2018 Mar 13;9:439. doi: 10.3389/fmicb.2018.00439 (PMC5859045; doi:10.3389/fmicb.2018.00439)

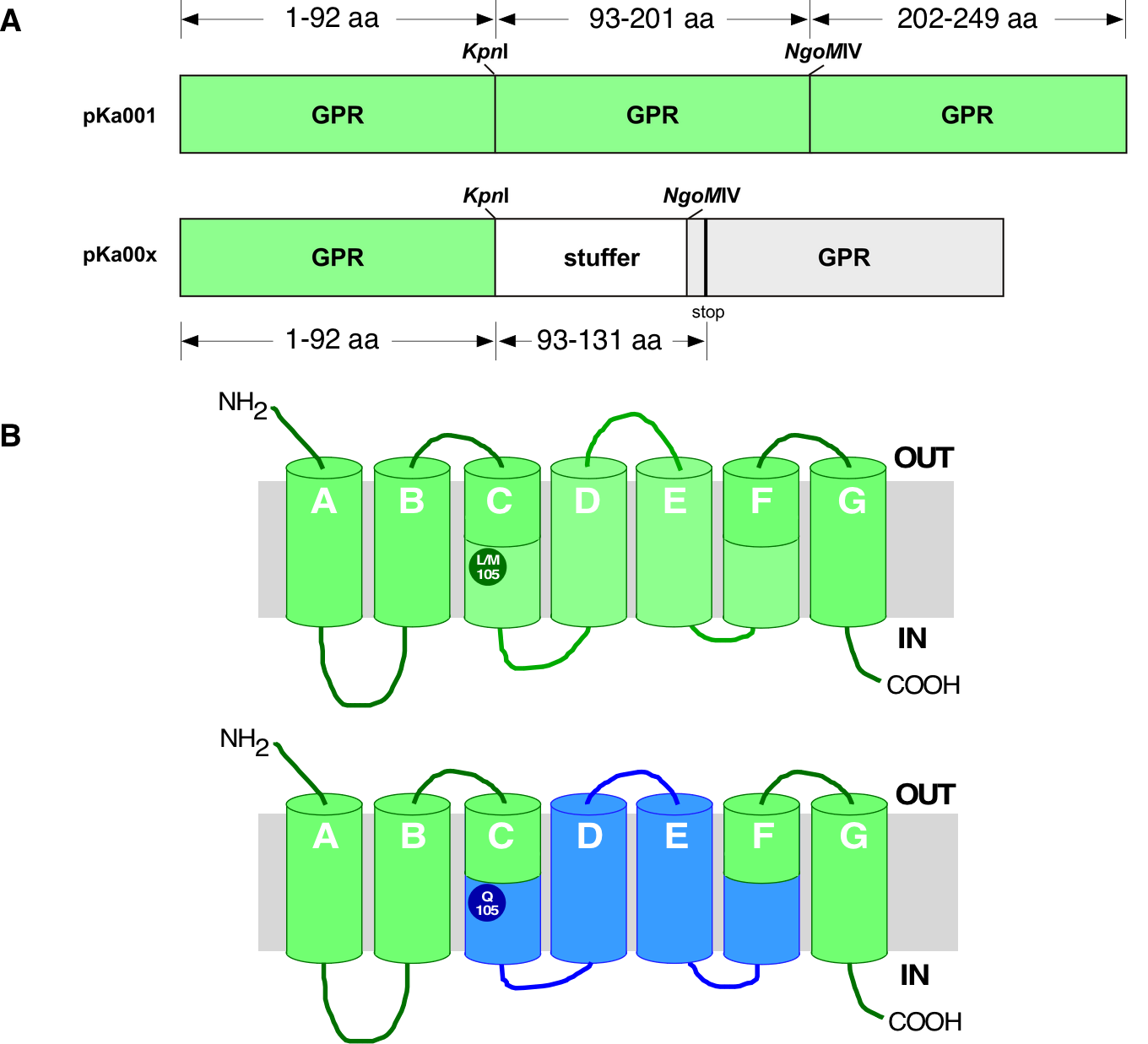

Supplement: FIGURE S1 — Chimeric construct for the expression of partial rhodopsin genes from the environment. (A) pKa001 is the original construct used for the expression of verified proteorhodopsin (PR) sequences in previous studies (GPR phenotype) compared to pKa00x harboring a short ORF disrupting random sequence (no phenotype) for background elimination during screening. (B) A schematic representation of the seven transmembranes of PR. The middle part is replaced by partial rhodopsins from the environment. The dominant spectral tuning residue is in the interchangeable region between helixes C and F. [file Image_1.tif]

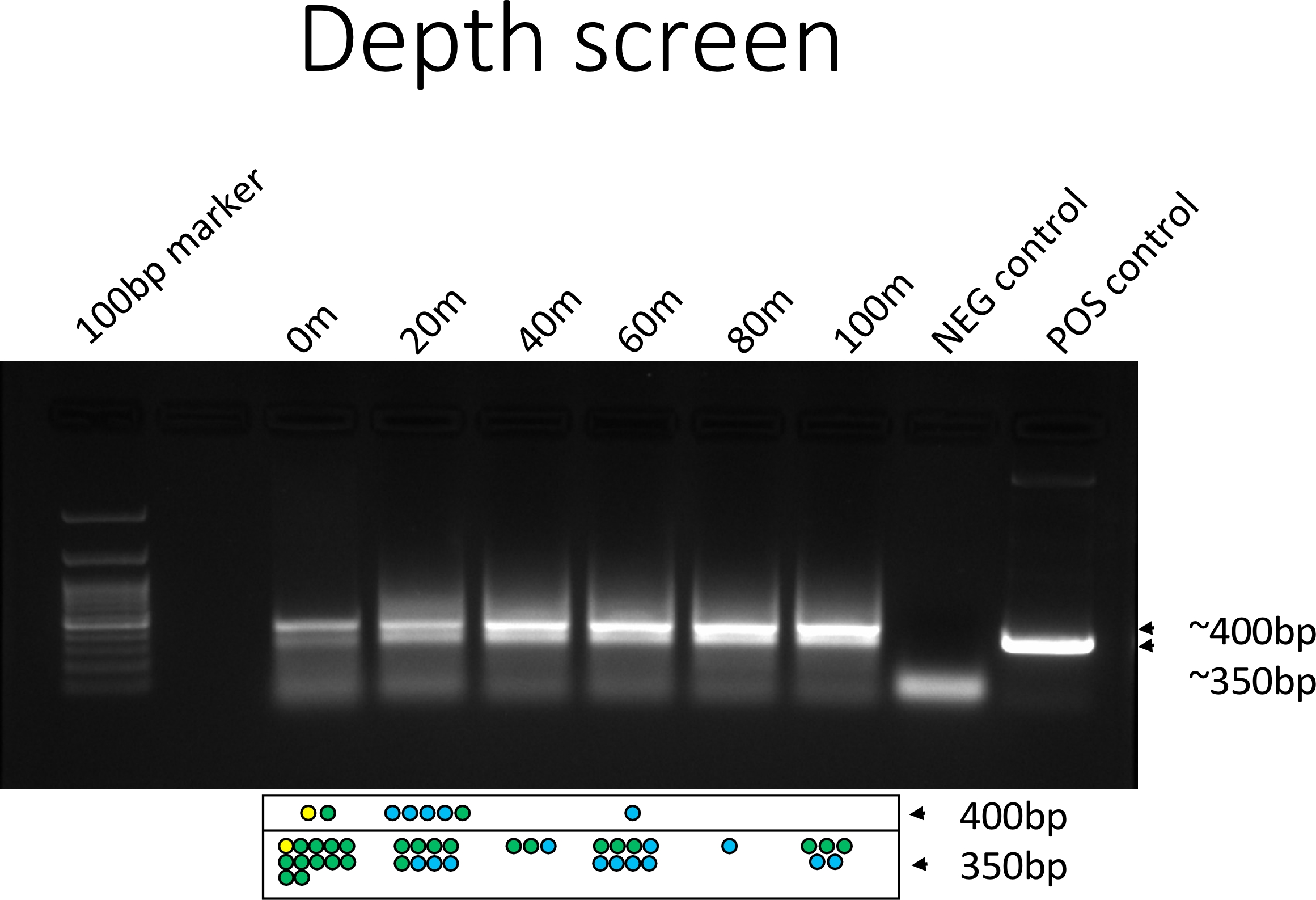

Supplement: FIGURE S2 — PCR resulted in PR amplification at all depths tested on March 3, 2014 at station A, the Red Sea. Primers used in this reaction are listed in Table 1. The reaction resulted in two bands of approximately 400 and 330 bp. The marker used in this 1% agarose gel is 100 bp DNA Ladder RTU (GeneDireX®). [file Image_2.JPEG]
