# Supplementary material for: The Use of a Chimeric Rhodopsin Vector for the Detection of New Proteorhodopsins Based on Color
Source: Front Microbiol. 2018 Mar 13;9:439. doi: 10.3389/fmicb.2018.00439 (PMC5859045; doi:10.3389/fmicb.2018.00439)
Supplement: Supplementary file 5 [file Data_Sheet_3.DOCX]

**Abbreviated** **protocol**

Centrifuge and observe phenotype

Plate into 96well plates (U shape) with induced retinal medium

Picking colonies into 96well plates LB-7% Glycerol

Plating on LB-Amp-Strep plates

Electroporation

Dialysis against DDW on VSWP 0.0025 filter 003j0.0025sekjnsdf

Ligation overnight at 4°C

Digest with NgoMIV and KpnI

Gel electrophoresis

Extraction of cut vector

Vector extraction (Qiagen only)

Phenol chloroform inactivation

Digest with NgoMIV and KpnI

Gel electrophoresis

PCR with degenerate primers

DNA extraction

Environmental/niche sampling

**Detailed protocol**

Preparations:

DH10B electrocompetent cells (30µl frozen at -80°C)

Streptomycin 25mg/ml stock

Ampicillin 100mg/ml stock

1. Plates: prepare 2L of LB and 2L of LB-agar. Antibiotics should be: amp 100µg/ml and streptomycin 25µg/ml final concentration. LB for 1L: 10gr tryptone, 5gr yeast extract, 10gr NaCl. For LB agar add 15gr agar for 1L and Autocalve.
2. DNA extraction protocol of your choosing. Here a standard phenol/chloroform protocol was used to extract DNA from 0.22 µm filter (after 20L filtration)
3. PCR: manufacturers’ protocol Takara Ex TaqTM polymerase (Takara-bio, Korea). The amplification program: an initial temperature stabilizing step at 98 °C for 5sec, followed by **40 cycles** at 98 °C for 10 sec (denaturation), 50°C for **90 sec** (**annealing**), and 72 °C for 120 sec (**elongation**). Finally, a post-elongation step at 72 °C for 120 sec was performed. The PCR products were separated by gel electrophoresis (1% agarose gel, 90 V, 35 min) and visualized by EtBr, against the marker 100 bp DNA Ladder. The desired product (approx. 330 bp) was excised from the gel and cleaned with the NucleoSpin® Gel and PCR Clean-up (Macherey-Nagel, Düren, Germany). Elute with app. 40 µl
4. Cutting the vector: DNA 5 µl (up to 1 µg! **Only Qiagen** extracted), NEB 1.1 buffer 5 µl, 1 µl KpnI, 1 µl NgoMIV, add DDW to total of 50 µl. 1-2h incubation at 37°C. Run on 1% agarose gel, 90 V, 35 min: insert excision (114bp) should be visible. Extract cut vector and elute in 40-50 µl.


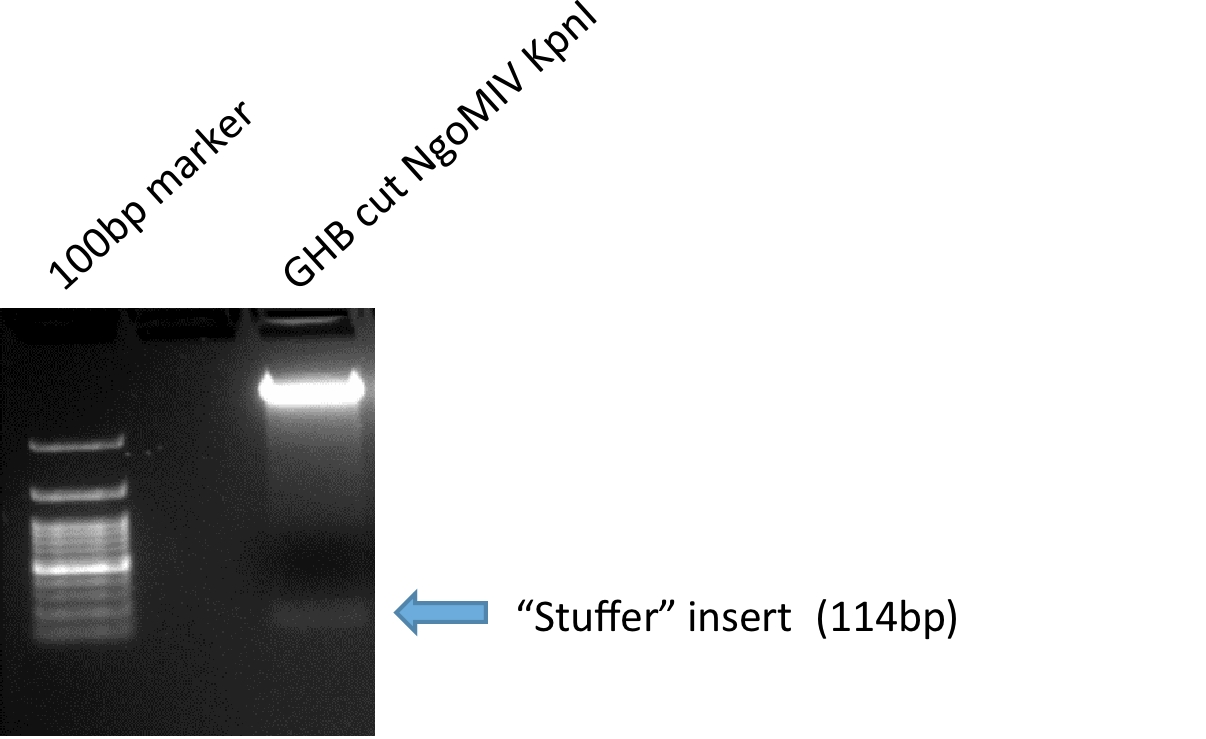


1. Cutting the PCR product: DNA: half of the elution, to allow a backup. NEB 1.1 buffer 5 µl, 1 µl KpnI, 1 µl NgoMIV, add DDW to total of 50 µl. Incubate 2-3h (no less) at 37°C. Phenol/chloroform extraction (extracts the enzymes and the small DNA fragments- **do not run gel instead,** since too little DNA will be left) . Add 25 µl phenol pH 8, 25 µl chloroform🡪vortex and spin down 2 min. Extract upper clear phase. Add 50 µl chloroform🡪vortex and spin 2 min. extract the upper clear phase. 10 min in 70°C, open for 3 min under the hood to allow the residual chloroform to evaporate safely.
2. Ligation: cut vector: 10 µl, cut insert: 7 µl, T4 buffer: 2 µl, T4 ligase 1 µl (total 20 µl) incubate either in PCR: 16°C or 4°C overnight. Inactivate by 10 min at 65°C.
3. Add 10 µl DDW, and try to electroporate into 30µl electrocompetent DH10B cells. If explodes, add more water or dialyze 1 hour on VSWP 0.0025 filter (Millipore, Inc) against DDW.
4. After dialysis, try 5 µl for electroporation.
5. Shake for 1h (tops) in 500 µl SOC/LB.
6. Plate 200 µl on amp-strep plates
7. Pick colonies into 96 well plates (for storage) (170 µl LB-7% glycerol in each well: for 20ml LB-7% glycerol add: 20 µl ampicillin, 20 µl streptomycin)
8. Pick colonies into 96 well plates (U shaped bottom, for pellet visualization) (170 LB µl in each well: for 20ml LB add: 10 µl amp, 30 µl IPTG (800mM stock) and 30 µl retinal (10mM stock). Cover with **breathable** tape (AeraSeal™ BS-25, EXCEL Scientific, USA) and **shake in incubator** at 250 rpm overnight.
9. Centrifuge with plate adjusted centrifuge. If plate centrifuge unavailable, leave plate still and untouched for several hours in RT.
10. Observe the colors of the precipitated cells and choose interesting colors for isolation and further study.
11. Obtaining spectra is possible in two ways: Whole cell concentrate or purified protein.

- Whole cell concentrate: Inoculate a fresh colony into 50ml with 25 µl amp, 75 µl IPTG and 75 µl retinal overnight in a 125ml flask, aerated. After 16-18 hours collect by centrifugation and resuspend in buffer A (50 mM Tris-HCl pH 8 and 5 mM MgCl_2_)- repeat twice. Resuspend in 1 ml buffer A and measure spectrum absorbance in a spectrophotometer. **Make note of buffers pH, since it can shift the absorbance dramatically**.
- The construct has a 6 His tag at the end of the protein, allowing purification and absorption spectra collection of clean protein, as described in Choi, 2013.
