# Supplementary material for: The Use of a Chimeric Rhodopsin Vector for the Detection of New Proteorhodopsins Based on Color
Source: Front Microbiol. 2018 Mar 13;9:439. doi: 10.3389/fmicb.2018.00439 (PMC5859045; doi:10.3389/fmicb.2018.00439)
Supplement: Supplementary file 6 [file Data_Sheet_4.DOCX]

**Rhodopsins found in this study- chimeric constructs:**

**Submitted in GeneBank under: KY963379-KY963416**

Legend:

- Green: ATG start codon
- Yellow: forward primer area, restriction site underlined KpnI
- Light blue: reverse primer area, restriction site underlined NgoMIV
- Dark blue: 6His tag
- Red: stop codon

>1_yellow_L_0m

ATGAAATTA

TTACTGATATTAGGTAGTGTTATTGCACTTCCTACATTTGCTGCAGGTGG

TGGTGACCTTGATGCTAGTGATTACACTGGTGTTTCTTTTTGGTTAGTTA

CTGCTGCTTTATTAGCATCTACTGTATTTTTCTTTGTTGAAAGAGATAGA

GTTTCTGCAAAATGGAAAACATCATTAACTGTATCTGGTCTTGTTACTGG

TATTGCTTTCTGGCATTACATGTACATGAGAGGGGTATGGATTGAAACTG

GTGATTCGCCAACTGTATTTAGGTACCTGGATTGGCTCTTAACAGTGCCG

CTGTTGATGGTAGAGTTCTATTTGATAATGTCAGCTGTCGGCAAAGTACC

GGGTCGAGTATTCTGGAATCTCTTAGGCGGTACCACCGTGATGCTGATCT

TTGGCTACATGGGTGAGACCGGCTCTATGGGTGTAGCCCCAGCATTCGCT

CTGTCAATGGCAGCATGGATCTATGTAATCTGGTACATCATGAAAGGTGA

AGCGAGCCAGGTTAATGCAAGCTTGGCCAATCCTAATGTGCAGAAAGCTT

ATAAGACGATGACTTTCCTTGTCACTGTCGGCTGGACCATTTACCCCGCC

GGCTATTTCACAGGTTACCTGATGGGTGACGGTGGATCAGCTCTTAACTT

AAACCTTATCTATAACCTTGCTGACTTTGTTAACAAGATTCTATTTGGTT

TAATTATATGGAATGTTGCTGTTAAAGAATCTTCTAATGCTCATCACCAC

CATCATCACTAA

>2_green_M_0m

ATGAAATTAT

TACTGATATTAGGTAGTGTTATTGCACTTCCTACATTTGCTGCAGGTGGT

GGTGACCTTGATGCTAGTGATTACACTGGTGTTTCTTTTTGGTTAGTTAC

TGCTGCTTTATTAGCATCTACTGTATTTTTCTTTGTTGAAAGAGATAGAG

TTTCTGCAAAATGGAAAACATCATTAACTGTATCTGGTCTTGTTACTGGT

ATTGCTTTCTGGCATTACATGTACATGAGAGGGGTATGGATTGAAACTGG

TGATTCGCCAACTGTATTTAGGTACCTGGACTGGATCTTAACAGTCCCGT

TGATGTGTGTTGAGTTTTATCTATTAACAAAAACTGGTTTGAGTAAAATG

GTTTCAGCTTCTGTTGTTATGCTTGTTACAGGTTATCTTGGCGAAGCATC

AGTTTGTTCAGTTTTTGGTACTCATAGCCCTGCAATATGGGGATTGATTA

GCGGTTTGGCGTATTTTTATATCGTTAATGAAGTGTACCAAGGCGATGTA

GCAAAAGCAGCAAAAAAAGCTGGTAAAAATATTCAAAATGCCAATTCACT

TCTGTTAAAGTTCGTTGTAATTGGCTGGTCAATCTATCCTGCCGGCTATT

TCACAGGTTACCTGATGGGTGACGGTGGATCAGCTCTTAACTTAAACCTT

ATCTATAACCTTGCTGACTTTGTTAACAAGATTCTATTTGGTTTAATTAT

ATGGAATGTTGCTGTTAAAGAATCTTCTAATGCTCATCACCACCATCATC

ACTAA

>3_blue_Q_20m

ATGAAATTAT

TACTGATATTAGGTAGTGTTATTGCACTTCCTACATTTGCTGCAGGTGGT

GGTGACCTTGATGCTAGTGATTACACTGGTGTTTCTTTTTGGTTAGTTAC

TGCTGCTTTATTAGCATCTACTGTATTTTTCTTTGTTGAAAGAGATAGAG

TTTCTGCAAAATGGAAAACATCATTAACTGTATCTGGTCTTGTTACTGGT

ATTGCTTTCTGGCATTACATGTACATGAGAGGGGTATGGATTGAAACTGG

TGATTCGCCAACTGTATTTAGGTACCTGGACTGGTTAATCACAGTGCCAT

TACAGATGGTAGAATTCTATTTAATTCTAGCAGCTATTGGTAAAGCAAAC

TCTGGAATGTTCTGGAGATTGTTAATCGGTTCATTAGTAATGCTAATTGG

TGGATACTTAGGTGAAGCAGGATATATTAATGCTACACTTGGTTTCATCA

TCGGAATGGCAGGTTGGGTATACATTCTTTATGAAGTATTCTCAGGTGAA

GCTGGAAAAGCTGCAGCGAAAAGTGGAAACAAAGCTCTTGTAACTGCTTT

TGGTGCAATGAGAATGATCGTTACTGTAGGATGGGGCATCTACCCCGCCG

GCTATTTCACAGGTTACCTGATGGGTGACGGTGGATCAGCTCTTAACTTA

AACCTTATCTATAACCTTGCTGACTTTGTTAACAAGATTCTATTTGGTTT

AATTATATGGAATGTTGCTGTTAAAGAATCTTCTAATGCTCATCACCACC

ATCATCACTAA

>4_blue_Q_20m

ATGAAATTA

TTACTGATATTAGGTAGTGTTATTGCACTTCCTACATTTGCTGCAGGTGG

TGGTGACCTTGATGCTAGTGATTACACTGGTGTTTCTTTTTGGTTAGTTA

CTGCTGCTTTATTAGCATCTACTGTATTTTTCTTTGTTGAAAGAGATAGA

GTTTCTGCAAAATGGAAAACATCATTAACTGTATCTGGTCTTGTTACTGG

TATTGCTTTCTGGCATTACATGTACATGAGAGGGGTATGGATTGAAACTG

GTGATTCGCCAACTGTATTTAGGTACCTGGACTGGCTAATCACAGTCCCA

CTGCAGGTCGTTGAATTCTACCTAATCCTCGCCGCTATCGGTGTGGGAAC

ATTCGTCATGTTCAGGAACCTCATGGCAGCATCCATCGTCATGCTAGTGG

CTGGATTCTTCGGTGAGTCCGGAGCAATGGACAACGTCATCGACCTGTCC

GCTGAGATCTGGTGGGTCATTGGAATGGCAGGATGGGGATACATCCTGTA

TGAGCTATGGTCCGGAGACGTCAAGGCGGCTTCAGAGACTGGAAGCCCAA

GCGTTCAGTACGCATTCAACTCAATGAGGTTGATTGTAACTTTCGGCTGG

AGCATATACCCCGCCGGCTATTTCACAGGTTACCTGATGGGTGACGGTGG

ATCAGCTCTTAACTTAAACCTTATCTATAACCTTGCTGACTTTGTTAACA

AGATTCTATTTGGTTTAATTATATGGAATGTTGCTGTTAAAGAATCTTCT

AATGCTCATCACCACCATCATCACTAA

>5_blue_Q_20m

ATGAAATTAT

TACTGATATTAGGTAGTGTTATTGCACTTCCTACATTTGCTGCAGGTGGT

GGTGACCTTGATGCTAGTGATTACACTGGTGTTTCTTTTTGGTTAGTTAC

TGCTGCTTTATTAGCATCTACTGTATTTTTCTTTGTTGAAAGAGATAGAG

TTTCTGCAAAATGGAAAACATCATTAACTGTATCTGGTCTTGTTACTGGT

ATTGCTTTCTGGCATTACATGTACATGAGAGGGGTATGGATTGAAACTGG

TGATTCGCCAACTGTATTTAGGTACCTGGACTGGTTAATCACTGTGCCTT

TACAGATGGTAGAATTTTACTTAATTCTAGCAGCTATCGGTAAAGCAAAC

TCTGGAATGTTCTGGAGATTGTTAATAGGTTCATTAGTAATGCTTATCGG

TGGATACTTAGGTGAGGCAGGATATATAAATGCTACACTTGGTTTCATCA

TCGGAATGGCAGGTTGGGTGTACATTCTTTACGAAGTATTCTCAGGTGAG

TCTGGAAGAGCTGCAGCTAAAAGTGGTAACAAAGCTCTTGTAACAGCTTT

TGGTGCTATGAGAATGATCGTTACAGTTGGCTGGAGCATCTATCCGGCCG

GCTATTTCACAGGTTACCTGATGGGTGACGGTGGATCAGCTCTTAACTTA

AACCTTATCTATAACCTTGCTGACTTTGTTAACAAGATTCTATTTGGTTT

AATTATATGGAATGTTGCTGTTAAAGAATCTTCTAATGCTCATCACCACC

ATCATCACTAA

>6_green_M_20m

ATGAAATTATT

ACTGATATTAGGTAGTGTTATTGCACTTCCTACATTTGCTGCAGGTGGTG

GTGACCTTGATGCTAGTGATTACACTGGTGTTTCTTTTTGGTTAGTTACT

GCTGCTTTATTAGCATCTACTGTATTTTTCTTTGTTGAAAGAGATAGAGT

TTCTGCAAAATGGAAAACATCATTAACTGTATCTGGTCTTGTTACTGGTA

TTGCTTTCTGGCATTACATGTACATGAGAGGGGTATGGATTGAAACTGGT

GATTCGCCAACTGTATTTAGGTACCTAGATTGGATCTTAACAGTCCCGTT

GATGTGTGTTGAGTTTTATCTATTAACAAAAACTGGTTTGAGTAAAATGG

TTTCAGCTTCTGTTGTTATGCTTGTTACAGGTTATCTTGGCGAAGCATCA

GTTTGTTCAGTTTTTGGTACTCATAGCCCTGCAATATGGGGATTGATTAG

CGGTTTGGCGTATTTTTATATCGTCAATGAAGTGTACCAAGGCGATGTAG

CAAAAGCAGCAAAAAAAGCTGGTAAAAATATTCAAAATGCCAACTCACTT

CTGTTAAAGTTCGTTGTAATTGGGTGGGGTATATACCCAGCCGGCTATTT

CACAGGTTACCTGATGGGTGACGGTGGATCAGCTCTTAACTTAAACCTTA

TCTATAACCTTGCTGACTTTGTTAACAAGATTCTATTTGGTTTAATTATA

TGGAATGTTGCTGTTAAAGAATCTTCTAATGCTCATCACCACCATCATCA

CTAA

>7_blue_Q_20m

ATGAAATTAT

TACTGATATTAGGTAGTGTTATTGCACTTCCTACATTTGCTGCAGGTGGT

GGTGACCTTGATGCTAGTGATTACACTGGTGTTTCTTTTTGGTTAGTTAC

TGCTGCTTTATTAGCATCTACTGTATTTTTCTTTGTTGAAAGAGATAGAG

TTTCTGCAAAATGGAAAACATCATTAACTGTATCTGGTCTTGTTACTGGT

ATTGCTTTCTGGCATTACATGTACATGAGAGGGGTATGGATTGAAACTGG

TGATTCGCCAACTGTATTTAGGTACCTTGACTGGTTAATCACAGTACCAC

TACAAGTCGTAGAATTCTACCTAATTCTAGCGGCAATCGGAGTGGGTACT

GCAATTATGTTCCAGAGACTACTTGGGGCATCTATTGTTATGCTGGTTGC

AGGATACTTCGGTGAATCCGGAGCTATGGACAGTACTCTGGAACTATCAC

CAACAGTCTGGTGGATAATCGGAATGGCCGGATGGGCATGGATTCTCTAC

GAACTATGGGCTGGAGAAGTCGGTGCAGCTGCAGAGAGCGGAAGCGCAGG

AGTCCAATATGGATTCAATGCAATGAAGTTGATTGTAACATTTGGTTGGG

CAATATATCCGGCCGGCTATTTCACAGGTTACCTGATGGGTGACGGTGGA

TCAGCTCTTAACTTAAACCTTATCTATAACCTTGCTGACTTTGTTAACAA

GATTCTATTTGGTTTAATTATATGGAATGTTGCTGTTAAAGAATCTTCTA

ATGCTCATCACCACCATCATCACTAA

>8_blue_Q_60m

ATGAAATTA

TTACTGATATTAGGTAGTGTTATTGCACTTCCTACATTTGCTGCAGGTGG

TGGTGACCTTGATGCTAGTGATTACACTGGTGTTTCTTTTTGGTTAGTTA

CTGCTGCTTTATTAGCATCTACTGTATTTTTCTTTGTTGAAAGAGATAGA

GTTTCTGCAAAATGGAAAACATCATTAACTGTATCTGGTCTTGTTACTGG

TATTGCTTTCTGGCATTACATGTACATGAGAGGGGTATGGATTGAAACTG

GTGATTCGCCAACTGTATTTAGGTACCTTGATTGGTTGCTAACAGTACCT

TTACAGATGGTAGAATTTTACCTAATTCTAGCTGCTGTTACTGTTGTAGC

TGGTTCTTTGTTCTGGCAATTACTTTTAGGTTCTTTAGTCATGTTGATTT

TTGGTTACATGGGTGAAGCTGGACTTATGGCTGCAATGCCTGCGTTTGTA

ATTGGAATGCTAGCTTGGCTTTGCATGATCTATGTACTTTACATGGGTGC

TGGTAAAGCTGCTGTTTCCTCAACAAGCGCGTCAGTTCAAACTGCATATA

ATTCAATGCTATTGATTATTGTTGTTGGATGGGTAATCTATCCGGCCGGC

TATTTCACAGGTTACCTGATGGGTGACGGTGGATCAGCTCTTAACTTAAA

CCTTATCTATAACCTTGCTGACTTTGTTAACAAGATTCTATTTGGTTTAA

TTATATGGAATGTTGCTGTTAAAGAATCTTCTAATGCTCATCACCACCAT

CATCACTAA

>9_green_M_0m

ATGAAATTA

TTACTGATATTAGGTAGTGTTATTGCACTTCCTACATTTGCTGCAGGTGG

TGGTGACCTTGATGCTAGTGATTACACTGGTGTTTCTTTTTGGTTAGTTA

CTGCTGCTTTATTAGCATCTACTGTATTTTTCTTTGTTGAAAGAGATAGA

GTTTCTGCAAAATGGAAAACATCATTAACTGTATCTGGTCTTGTTACTGG

TATTGCTTTCTGGCATTACATGTACATGAGAGGGGTATGGATTGAAACTG

GTGATTCGCCAACTGTATTTAGGTACCTGGATTGGATCTTAACAGTCCCG

TTGATGTGTGTTGAATTTTATCTATTAACAAAAACTGGTTTGAGTAAAAT

GGTTTCAGCTTCTGTTGTTATGCTTGTTACAGGTTATCTTGGCGAAGCAT

CAGTTTGTTCAGTTTTTGGTACTCATAGCCCTGCAATATGGGGATTGATT

AGCGGTTTGGCGTATTTTTATATCGTCAATGAAGTGTACCAAGGCGATGT

AGCAAAAGCAGCAAAAAAAGCTGGTAAAAATATTCAAAATGCCAACTCAC

TTCTGTTAAAGTTCGTTGTAATTGGCTGGGGCATCTATCCTGCCGGCTAT

TTCACAGGTTACCTGATGGGTGACGGTGGATCAGCTCTTAACTTAAACCT

TATCTATAACCTTGCTGACTTTGTTAACAAGATTCTATTTGGTTTAATTA

TATGGAATGTTGCTGTTAAAGAATCTTCTAATGCTCATCACCACCATCAT

CACTAA

>10_yellow_L_0m

ATGAAATTATT

ACTGATATTAGGTAGTGTTATTGCACTTCCTACATTTGCTGCAGGTGGTG

GTGACCTTGATGCTAGTGATTACACTGGTGTTTCTTTTTGGTTAGTTACT

GCTGCTTTATTAGCATCTACTGTATTTTTCTTTGTTGAAAGAGATAGAGT

TTCTGCAAAATGGAAAACATCATTAACTGTATCTGGTCTTGTTACTGGTA

TTGCTTTCTGGCATTACATGTACATGAGAGGGGTATGGATTGAAACTGGT

GATTCGCCAACTGTATTTAGGTACCTTGACTGGTTACTTACAGTGCCACT

CTTGATGGTAGAGTTCTACCTAATTATGTCGGCAGTAGGGAAGGTGCCTG

GTCGTGTTTTCTGGCACTTGTTAGTAGGCTCTACGCTTATGCTGTTATTC

GGTTATGCAGGCGAGACCGGAATGATGGACGTTGGCTTGGCCTTCTGGCT

GGCAATGGCGGCTTGGATATATATAATCTGGTACATCATTAAGGGTGAAG

CAAGTCAGATTAACGCAAGCCTAGCAAATGCCAACGTGCAAAAAGCCTAT

AAGGCAATGACCTTCTTGGTCACTGTCGGGTGGACTATATACCCTGCCGG

CTATTTCACAGGTTACCTGATGGGTGACGGTGGATCAGCTCTTAACTTAA

ACCTTATCTATAACCTTGCTGACTTTGTTAACAAGATTCTATTTGGTTTA

ATTATATGGAATGTTGCTGTTAAAGAATCTTCTAATGCTCATCACCACCA

TCATCACTAA

>11_green_M_0m

ATGAAATT

ATTACTGATATTAGGTAGTGTTATTGCACTTCCTACATTTGCTGCAGGTG

GTGGTGACCTTGATGCTAGTGATTACACTGGTGTTTCTTTTTGGTTAGTT

ACTGCTGCTTTATTAGCATCTACTGTATTTTTCTTTGTTGAAAGAGATAG

AGTTTCTGCAAAATGGAAAACATCATTAACTGTATCTGGTCTTGTTACTG

GTATTGCTTTCTGGCATTACATGTACATGAGAGGGGTATGGATTGAAACT

GGTGATTCGCCAACTGTATTTAGGTACCTGGATTGGATCTTAACAGTCCC

GTTGATGTGTGTTGAGTTTTATCTATTAACAAAAACTGGTTTGAGTAAAA

TGGTTTCAGCTTCTGTTGTTATGCTTGTTACAGGTTATCTTGGCGAAGCA

TCAGTTTGTTCAGTTTTTGGTACTCATAGCCCTGCAATATGGGGATTGAT

TAGCGGTTTGGCGTATTTTTATATCGTCAATGAAGTGTACCAAGGCGATG

TAGCAAAAGCAGCAAAAAAAGCTGGTAAAAATATTCAAAATGCCAACTCA

CTTCTGTTAAAGTTCGTTGTAATTGGGTGGTCCATCTATCCTGCCGGCTA

TTTCACAGGTTACCTGATGGGTGACGGTGGATCAGCTCTTAACTTAAACC

TTATCTATAACCTTGCTGACTTTGTTAACAAGATTCTATTTGGTTTAATT

ATATGGAATGTTGCTGTTAAAGAATCTTCTAATGCTCATCACCACCATCA

TCACTAA

>12_green_M_0m

ATGAAATTAT

TACTGATATTAGGTAGTGTTATTGCACTTCCTACATTTGCTGCAGGTGGT

GGTGACCTTGATGCTAGTGATTACACTGGTGTTTCTTTTTGGTTAGTTAC

TGCTGCTTTATTAGCATCTACTGTATTTTTCTTTGTTGAAAGAGATAGAG

TTTCTGCAAAATGGAAAACATCATTAACTGTATCTGGTCTTGTTACTGGT

ATTGCTTTCTGGCATTACATGTACATGAGAGGGGTATGGATTGAAACTGG

TGATTCGCCAACTGTATTTAGGTACCTCGATTGGATCTTAACAGTCCCGT

TGATGTGTGTTGAGTTTTATCTATTAACAAAAACTGGTTTGAGTAAAATG

GTTTCAGCTTCTGTTGTTATGCTTGTTACAGGTTATCTTGGCGAAGCATC

AGTTTGTTCAGTTTTTGGTACTCATAGCCCTGCAATATGGGGATTGATTA

GCGGTTTGGCGTATTTTTATATCGTCAATGAAGTGTACCAAGGCGATGTA

GCAAAAGCAGCAAAAAAAGCTGGTAAAAATATTCAAAATGCCAATTCGCT

TCTGTTAAAGTTCGTTGTAATTGGGTGGACCATATACCCCGCCGGCTATT

TCACAGGTTACCTGATGGGTGACGGTGGATCAGCTCTTAACTTAAACCTT

ATCTATAACCTTGCTGACTTTGTTAACAAGATTCTATTTGGTTTAATTAT

ATGGAATGTTGCTGTTAAAGAATCTTCTAATGCTCATCACCACCATCATC

ACTAA

>13_green_M_0m
ATGAAATTATT

ACTGATATTAGGTAGTGTTATTGCACTTCCTACATTTGCTGCAGGTGGTG

GTGACCTTGATGCTAGTGATTACACTGGTGTTTCTTTTTGGTTAGTTACT

GCTGCTTTATTAGCATCTACTGTATTTTTCTTTGTTGAAAGAGATAGAGT

TTCTGCAAAATGGAAAACATCATTAACTGTATCTGGTCTTGTTACTGGTA

TTGCTTTCTGGCATTACATGTACATGAGAGGGGTATGGATTGAAACTGGT

GATTCGCCAACTGTATTTAGGTACCTTGATTGGATCTTAACAGTCCCGTT

GATGTGTGTTGAGTTTTATCTATTAACAAAAACTGGTTTGAGTAAAATGG

TTTCAGCTTCTGTTGTTATGCTTGTTACAGGTTATCTTGGCGAAGCATCA

GTTTGTTCAGTTTTTGGTACTCATAGCCCTGCAATATGGGGATTGATTAG

CGGTTTGGCGTATTTTTATATCGTCAATGAAGTGTACCAAGGCGATGTAG

CAAAAGCAGCAAAAAAAGCTGGTAAAAATATTCAAAATGCCAACTCACTT

CTGTTAAAGTTCGTTGTAATTGGGTGGGGCATATACCCCGCCGGCTATTT

CACAGGTTACCTGATGGGTGACGGTGGATCAGCTCTTAACTTAAACCTTA

TCTATAACCTTGCTGACTTTGTTAACAAGATTCTATTTGGTTTAATTATA

TGGAATGTTGCTGTTAAAGAATCTTCTAATGCTCATCACCACCATCATCA

CTAA

>14_green_M_0m

ATGAAATTATT

ACTGATATTAGGTAGTGTTATTGCACTTCCTACATTTGCTGCAGGTGGTG

GTGACCTTGATGCTAGTGATTACACTGGTGTTTCTTTTTGGTTAGTTACT

GCTGCTTTATTAGCATCTACTGTATTTTTCTTTGTTGAAAGAGATAGAGT

TTCTGCAAAATGGAAAACATCATTAACTGTATCTGGTCTTGTTACTGGTA

TTGCTTTCTGGCATTACATGTACATGAGAGGGGTATGGATTGAAACTGGT

GATTCGCCAACTGTATTTAGGTACCTGGATTGGATCTTAACAGTCCCGTT

GATGTGTGTTGAGTTTTATCTATTAACAAAAACTGGTTTGAGTAAAATGG

TTTCAGCTTCTGTTGTTATGCTTGTTACAGGTTATCTTGGCGAAGCATCA

GTTTGTTCAGTTTTTGGTACTCATAGCCCTGCAATATGGGGATTGATTAG

CGGTTTGGCGTATTTTTATATCGTCAATGAAGTGTACCAAGGCGATGTAG

CAAAAGCAGCAAAAAAAGCTGGTAAAAATATTCAAAATGCCAACTCACTT

CTGTTAAAGTTCGTTGTAATTGGGTGGGCAATCTACCCGGCCGGCTATTT

CACAGGTTACCTGATGGGTGACGGTGGATCAGCTCTTAACTTAAACCTTA

TCTATAACCTTGCTGACTTTGTTAACAAGATTCTATTTGGTTTAATTATA

TGGAATGTTGCTGTTAAAGAATCTTCTAATGCTCATCACCACCATCATCA

CTAA

>15_green_M_0m

ATGAAATTA

TTACTGATATTAGGTAGTGTTATTGCACTTCCTACATTTGCTGCAGGTGG

TGGTGACCTTGATGCTAGTGATTACACTGGTGTTTCTTTTTGGTTAGTTA

CTGCTGCTTTATTAGCATCTACTGTATTTTTCTTTGTTGAAAGAGATAGA

GTTTCTGCAAAATGGAAAACATCATTAACTGTATCTGGTCTTGTTACTGG

TATTGCTTTCTGGCATTACATGTACATGAGAGGGGTATGGATTGAAACTG

GTGATTCGCCAACTGTATTTAGGTACCTGGATTGGATCTTAACAGTCCCG

TTGATGTGTGTTGAGTTTTATCTATTAACAAAAACTGGTTTGAGTAAAAT

GGTTTCAGCTTCTGTTGTTATGCTTGTTACAGGTTATCTTGGCGAAGCAT

CAGTTTGTTCAGTTTTTGGTACTCATAGCCCTGCAATATGGGGATTGATT

AGCGGTTTGGCGTATTTTTATATCGTCAATGAAGTGTACCAAGGCGATGT

AGCAAAAGCAGCAAAAAAAGCTGGTAAAAATATTCAAAATGCCAACTCAC

TTCTGTTAAAGTTCGTTGTAATTGGCTGGTGCATCTATCCGGCCGGCTAT

TTCACAGGTTACCTGATGGGTGACGGTGGATCAGCTCTTAACTTAAACCT

TATCTATAACCTTGCTGACTTTGTTAACAAGATTCTATTTGGTTTAATTA

TATGGAATGTTGCTGTTAAAGAATCTTCTAATGCTCATCACCACCATCAT

CACTAA

>16_green_M_0m

ATGAAATTAT

TACTGATATTAGGTAGTGTTATTGCACTTCCTACATTTGCTGCAGGTGGT

GGTGACCTTGATGCTAGTGATTACACTGGTGTTTCTTTTTGGTTAGTTAC

TGCTGCTTTATTAGCATCTACTGTATTTTTCTTTGTTGAAAGAGATAGAG

TTTCTGCAAAATGGAAAACATCATTAACTGTATCTGGTCTTGTTACTGGT

ATTGCTTTCTGGCATTACATGTACATGAGAGGGGTATGGATTGAAACTGG

TGATTCGCCAACTGTATTTAGGTACCTGGATTGGATCTTAACAGTCCCGT

TGATGTGTGTTGAGTTTTATCTATTAACAAAAACTGGTTTGAGTAAAATG

GTTACAGCTTCTGTTGTTATGCTTGTTACAGGTTATCTTGGCGAAGCATC

AGTTTGTTCAGTTTTTGGTACTCATAGCCCTGCAATATGGGGATTGATTA

GCGGTTTGGCGTATTTTTATATCGTCAATGAAGTGTACCAAGGCGATGTA

GCAAAAGCAGCAAAAAAAGCTGGTAAAAATATTCAAAATGCCAATTCGCT

TCTGTTAAAGTTCGTTGTAATTGGATGGGTCATCTACCCCGCCGGCTATT

TCACAGGTTACCTGATGGGTGACGGTGGATCAGCTCTTAACTTAAACCTT

ATCTATAACCTTGCTGACTTTGTTAACAAGATTCTATTTGGTTTAATTAT

ATGGAATGTTGCTGTTAAAGAATCTTCTAATGCTCATCACCACCATCATC

ACTAA

>17_green_M_0m

ATGAAAT

TATTACTGATATTAGGTAGTGTTATTGCACTTCCTACATTTGCTGCAGGT

GGTGGTGACCTTGATGCTAGTGATTACACTGGTGTTTCTTTTTGGTTAGT

TACTGCTGCTTTATTAGCATCTACTGTATTTTTCTTTGTTGAAAGAGATA

GAGTTTCTGCAAAATGGAAAACATCATTAACTGTATCTGGTCTTGTTACT

GGTATTGCTTTCTGGCATTACATGTACATGAGAGGGGTATGGATTGAAAC

TGGTGATTCGCCAACTGTATTTAGGTACCTGGATTGGATCTTAACAGTCC

CGTTGATGTGTGTTGAGTTTTATCTATTAACAAAAACTGGTTTGAGTAAA

ATGGTTTCAGCTTCTGTTGTTATGCTTGTTACAGGTTATCTTGGCGAAGC

ATCAGTTTGTTCAGTTTTTGGTACTCATAGCCCTGCAATATGGGGATTGA

TTAGCGGTTTGGCGTATTTTTATATCGTCAATGAAGTGTACCAAGGCGAT

GTAGCAAAAGCAGCAAAAAAAGCTGGTAAAAATATTCAAAATGCCAACTC

ACTTCTGTTAAAGTTCGTTGTAATTGGTTGGGCTATCTATCCAGCCGGCT

ATTTCACAGGTTACCTGATGGGTGACGGTGGATCAGCTCTTAACTTAAAC

CTTATCTATAACCTTGCTGACTTTGTTAACAAGATTCTATTTGGTTTAAT

TATATGGAATGTTGCTGTTAAAGAATCTTCTAATGCTCATCACCACCATC

ATCACTAA

>18_green_M_0m

ATGAAATTA

TTACTGATATTAGGTAGTGTTATTGCACTTCCTACATTTGCTGCAGGTGG

TGGTGACCTTGATGCTAGTGATTACACTGGTGTTTCTTTTTGGTTAGTTA

CTGCTGCTTTATTAGCATCTACTGTATTTTTCTTTGTTGAAAGAGATAGA

GTTTCTGCAAAATGGAAAACATCATTAACTGTATCTGGTCTTGTTACTGG

TATTGCTTTCTGGCATTACATGTACATGAGAGGGGTATGGATTGAAACTG

GTGATTCGCCAACTGTATTTAGGTACCTGGACTGGATACTCACGGTCCCC

CTAATGTGCGTTGAGTTTTATCTCATAACGAAGAAAGCCGGCGCTAAAAT

TGGGCTGCTATGGAAGCTCATCGCAGCATCGGTCTTTATGCTCGTGACTG

GTTACTTCGGCGAAACCGTTTACAGAGACGCCAGTGTTTTTTGGGGTGTC

GTATCTGGTGCTGCTTACTTTTATATTGTTTACCTCATTTGGTTCGGGGA

AGTGGCCAAGCTCGCCACCGACGCAGGGCCACAGGTGGCAAAGGCAAATC

GGGTGTTGGCTTGGTTCGTGTTCGTGGGCTGGGGCATCTATCCGGCCGGC

TATTTCACAGGTTACCTGATGGGTGACGGTGGATCAGCTCTTAACTTAAA

CCTTATCTATAACCTTGCTGACTTTGTTAACAAGATTCTATTTGGTTTAA

TTATATGGAATGTTGCTGTTAAAGAATCTTCTAATGCTCATCACCACCAT

CATCACTAA

>19_green_M_0m

ATGAAATTAT

TACTGATATTAGGTAGTGTTATTGCACTTCCTACATTTGCTGCAGGTGGT

GGTGACCTTGATGCTAGTGATTACACTGGTGTTTCTTTTTGGTTAGTTAC

TGCTGCTTTATTAGCATCTACTGTATTTTTCTTTGTTGAAAGAGATAGAG

TTTCTGCAAAATGGAAAACATCATTAACTGTATCTGGTCTTGTTACTGGT

ATTGCTTTCTGGCATTACATGTACATGAGAGGGGTATGGATTGAAACTGG

TGATTCGCCAACTGTATTTAGGTACCTGGATTGGATCTTAACAGTCCCGT

TGATGTGTGTTGAGTTTTATCTATTAACAAAAACTGGTTTGAGTAAAATG

GTTTCAGCTTCTGTTGTTATGCTTGTTACAGGTTATCTTGGCGAAGCATC

AGTTTGTTCAGTTTTTGGTACTCATAGCCCTGCAATATGGGGATTGATTA

GCGGTTTGGCGTATTTTTATATCGTCAATGAAGTGTACCAAGGCGATGTA

GCAAAAGCAGCAAAAAAAGCTGGTAAAAATATTCAAAATGCCAACTCACT

TCTGTTAAAGTTCGTTGTAATTGGCTGGACCATCTATCCGGCCGGCTATT

TCACAGGTTACCTGATGGGTGACGGTGGATCAGCTCTTAACTTAAACCTT

ATCTATAACCTTGCTGACTTTGTTAACAAGATTCTATTTGGTTTAATTAT

ATGGAATGTTGCTGTTAAAGAATCTTCTAATGCTCATCACCACCATCATC

ACTAA

>20_green_M_0m

ATGAAATTAT

TACTGATATTAGGTAGTGTTATTGCACTTCCTACATTTGCTGCAGGTGGT

GGTGACCTTGATGCTAGTGATTACACTGGTGTTTCTTTTTGGTTAGTTAC

TGCTGCTTTATTAGCATCTACTGTATTTTTCTTTGTTGAAAGAGATAGAG

TTTCTGCAAAATGGAAAACATCATTAACTGTATCTGGTCTTGTTACTGGT

ATTGCTTTCTGGCATTACATGTACATGAGAGGGGTATGGATTGAAACTGG

TGATTCGCCAACTGTATTTAGGTACCTGGACTGGATCTTAACAGTCCCGT

TGATGTGTGTTGAGTTTTATCTATTAACAAAAACTGGTTTGAGTAAAATG

GTTTCAGCTTCTGTTGTTATGCTTGTTACAGGTTATCTTGGCGAAGCATC

AGTTTGTTCAGTTTTTGGTACTCATAGCCCTGCAATATGGGGATTGATTA

GCGGTTTGGCGTATTTTTATATCGTCAATGAAGTGTACCAAGGTGATGTA

GCAAAAGCAGCAAAAAAAGCTGGTAAAAATATTCAAAATGCCAACTCACT

TCTGTTAAAGTTCGTTGTAATTGGCTGGGCCATTTACCCGGCCGGCTATT

TCACAGGTTACCTGATGGGTGACGGTGGATCAGCTCTTAACTTAAACCTT

ATCTATAACCTTGCTGACTTTGTTAACAAGATTCTATTTGGTTTAATTAT

ATGGAATGTTGCTGTTAAAGAATCTTCTAATGCTCATCACCACCATCATC

ACTAA

>21_green_M_20m

ATGAAATTATT

ACTGATATTAGGTAGTGTTATTGCACTTCCTACATTTGCTGCAGGTGGTG

GTGACCTTGATGCTAGTGATTACACTGGTGTTTCTTTTTGGTTAGTTACT

GCTGCTTTATTAGCATCTACTGTATTTTTCTTTGTTGAAAGAGATAGAGT

TTCTGCAAAATGGAAAACATCATTAACTGTATCTGGTCTTGTTACTGGTA

TTGCTTTCTGGCATTACATGTACATGAGAGGGGTATGGATTGAAACTGGT

GATTCGCCAACTGTATTTAGGTACCTGGACTGGATCTTAACAGTCCCGTT

GATGTGTGTTGAGTTTTATCTATTAACAAAAACTGGTTTGAGTAAAATGG

TTTCAGCTTCTGTTGTTATGCTTGTTACAGGTTATCTTGGTGAAGCATCA

GTTTGTTCAGTTTTTGGTACTCATAGCCCTGCAATATGGGGATTGATTAG

CGGTTTGGCGTATTTTTATATCGTCAATGAAGTGTACCAAGGCGATGTAG

CAAAAGCAGCAAAAAAAGCTGGTAAAAATATTCAAAATGCCAACTCACTT

CTGTTAAAGTTCGTTGTAATTGGATGGGCAATTTACCCCGCCGGCTATTT

CACAGGTTACCTGATGGGTGACGGTGGATCAGCTCTTAACTTAAACCTTA

TCTATAACCTTGCTGACTTTGTTAACAAGATTCTATTTGGTTTAATTATA

TGGAATGTTGCTGTTAAAGAATCTTCTAATGCTCATCACCACCATCATCA

CTAA

>22_green_M_20m

ATGAAATTA

TTACTGATATTAGGTAGTGTTATTGCACTTCCTACATTTGCTGCAGGTGG

TGGTGACCTTGATGCTAGTGATTACACTGGTGTTTCTTTTTGGTTAGTTA

CTGCTGCTTTATTAGCATCTACTGTATTTTTCTTTGTTGAAAGAGATAGA

GTTTCTGCAAAATGGAAAACATCATTAACTGTATCTGGTCTTGTTACTGG

TATTGCTTTCTGGCATTACATGTACATGAGAGGGGTATGGATTGAAACTG

GTGATTCGCCAACTGTATTTAGGTACCTTGATTGGATCTTAACAGTCCCG

TTGATGTGTGTTGAGTTTTATCTATTAACAAAAACTGGTTTGAGTAAAAT

GGTTTCAGCTTCTGTTGTTATGCTTGTTACAGGTTATCTTGGCGAAGCAT

CAGTTTGTTCAGTTTTTGGTACTCATAGCCCTGCAATATGGGGATTGATT

AGCGGTTTGGCGTATTTTTATATCGTCAATGAAGTGTACCAAGGCGATGT

AGCAAAAGCAGCAAAAAAAGCTGGTAAAAATATTCAAAATGCCAATTCGC

TTCTGTTAAAGTTCGTTGTAATTGGTTGGACGATTTACCCTGCCGGCTAT

TTCACAGGTTACCTGATGGGTGACGGTGGATCAGCTCTTAACTTAAACCT

TATCTATAACCTTGCTGACTTTGTTAACAAGATTCTATTTGGGTTAATTA

TATGGAATGTTGCTGTTAAAGAATCTTCTAATGCTCATCACCACCATCAT

CACTAA

>23_blue_Q_20m

ATGAAATTATT

ACTGATATTAGGTAGTGTTATTGCACTTCCTACATTTGCTGCAGGTGGTG

GTGACCTTGATGCTAGTGATTACACTGGTGTTTCTTTTTGGTTAGTTACT

GCTGCTTTATTAGCATCTACTGTATTTTTCTTTGTTGAAAGAGATAGAGT

TTCTGCAAAATGGAAAACATCATTAACTGTATCTGGTCTTGTTACTGGTA

TTGCTTTCTGGCATTACATGTACATGAGAGGGGTATGGATTGAAACTGGT

GATTCGCCAACTGTATTTAGGTACCTTGACTGGTTAATCACTGTGCCATT

ACAGATGGTAGAATTCTATTTAATTCTAGCAGCTATTGGTAAAGCAAACT

CTGGAATGTTCTGGAGATTGTTAATTGGTTCATTAGTAATGCTTATCGGT

GGATACTTAGGTGAAGCAGGATATATTAATGCTACACTAGGTTTCATCAT

CGGAATGGCAGGTTGGGTATACATTCTTTATGAAGTATTCTCAGGTGAAG

CTGGTAAAGCTGCACAGAAGAGTGGTAACAAAGCTCTTGTAACTGCATTC

GGAGCAATGAGAATGATCGTTACAGTAGGGTGGGGCATATATCCTGCCGG

CTATTTCACAGGTTACCTGATGGGTGACGGTGGATCAGCTCTTAACTTAA

ACCTTATCTATAACCTTGCTGACTTTGTTAACAAGATTCTATTTGGTTTA

ATTATATGGAATGTTGCTGTTAAAGAATCTTCTAATGCTCATCACCACCA

TCATCACTAA

>24_blue_Q_20m

ATGAAATTA

TTACTGATATTAGGTAGTGTTATTGCACTTCCTACATTTGCTGCAGGTGG

TGGTGACCTTGATGCTAGTGATTACACTGGTGTTTCTTTTTGGTTAGTTA

CTGCTGCTTTATTAGCATCTACTGTATTTTTCTTTGTTGAAAGAGATAGA

GTTTCTGCAAAATGGAAAACATCATTAACTGTATCTGGTCTTGTTACTGG

TATTGCTTTCTGGCATTACATGTACATGAGAGGGGTATGGATTGAAACTG

GTGATTCGCCAACTGTATTTAGGTACCTCGACTGGTTAATCACAGTACCA

CTACAAGTCGTAGAATTCTACCTAATTCTAGCGGCAATCGGAGTGGGTAC

TGCAATTATGTTCCAGAGACTACTTGGGGCATCTATTGTTATGCTGGTTG

CAGGATACTTCGGTGAATCCGGAGCTATGGACAGTACTCTGGAACTATCA

CCAACAGTCTGGTGGATAATCGGAATGGCCGGATGGGCATGGATTCTCTA

CGAACTATGGGCTGGAGAAGTCGGTGCAGCTGCAGAGAGCGGAAGCGCAG

GAGTCCAATATGGATTCAATGCAATGAAGTTGATTGTAACATTTGGGTGG

ACAATCTATCCCGCCGGCTATTTCACAGGTTACCTGATGGGTGACGGTGG

ATCAGCTCTTAACTTAAACCTTATCTATAACCTTGCTGACTTTGTTAACA

AGATTCTATTTGGTTTAATTATATGGAATGTTGCTGTTAAAGAATCTTCT

AATGCTCATCACCACCATCATCACTAA

>25_green_M_20m

ATGAAATTAT

TACTGATATTAGGTAGTGTTATTGCACTTCCTACATTTGCTGCAGGTGGT

GGTGACCTTGATGCTAGTGATTACACTGGTGTTTCTTTTTGGTTAGTTAC

TGCTGCTTTATTAGCATCTACTGTATTTTTCTTTGTTGAAAGAGATAGAG

TTTCTGCAAAATGGAAAACATCATTAACTGTATCTGGTCTTGTTACTGGT

ATTGCTTTCTGGCATTACATGTACATGAGAGGGGTATGGATTGAAACTGG

TGATTCGCCAACTGTATTTAGGTACCTGGATTGGATATTAACTGTACCAT

TGATGTGTGTTGAATTCTATCTTCTTACAAACGTTGGTTTGAAAAAAATG

GTTACAGCATCAGTAGTTATGTTAATAACCGGGTATTTTGGAGAAGCAGG

TCTTTTTCCTATTGGAAATATAGGTCCAGCATTTTGGGGCCTCTTGAGTG

GACTAGCGTATTTTTATATTGTAAATGAAGTTTACCGTGGTGATATAGCA

AAAGCAGCTGCAAAAGCAGGTGGAAAAATAGTAAATGCAAATTCACTGCT

ATTAAAATTTATCGTAATTGGTTGGACCATATATCCCGCCGGCTATTTCA

CAGGTTACCTGATGGGTGACGGTGGATCAGCTCTTAACTTAAACCTTATC

TATAACCTTGCTGACTTTGTTAACAAGATTCTATTTGGGTTAATTATATG

GAATGTTGCTGTTAAAGAATCTTCTAATGCTCATCACCACCATCATCACT

AA

>26_green_M_20m

ATGAAATTATTA

CTGATATTAGGTAGTGTTATTGCACTTCCTACATTTGCTGCAGGTGGTGG

TGACCTTGATGCTAGTGATTACACTGGTGTTTCTTTTTGGTTAGTTACTG

CTGCTTTATTAGCATCTACTGTATTTTTCTTTGTTGAAAGAGATAGAGTT

TCTGCAAAATGGAAAACATCATTAACTGTATCTGGTCTTGTTACTGGTAT

TGCTTTCTGGCATTACATGTACATGAGAGGGGTATGGATTGAAACTGGTG

ATTCGCCAACTGTATTTAGGTACCTTGATTGGATCTTAACAGTCCCGTTG

ATGTGTGTTGAGTTTTATCTATTAACAAAAACTGGTTTGAGTAAAATGGT

TTCAGCTTCTGTTGTTATGCTTGTTACAGGTTATCTTGGCGAAGCATCAG

TTTGTTCAGTTTTTGGTACTCATAGCCCTGCAATATGGGGATTGATTAGC

GGTTTGGCGTATTTTTATATCGTCAATGAAGTGTACCAAGGCGATGTAGC

AAAAGCAGCAAAAAAAGCTGGTAAAAATATTCAAAATGCCAATTCGCTTC

TGTTAAAGTTCGTTGTAATTGGGTGGGCCATCTATCCCGCCGGCTATTTC

ACAGGTTACCTGATGGGTGACGGTGGATCAGCTCTTAACTTAAACCTTAT

CTATAACCTTGCTGACTTTGTTAACAAGATTCTATTTGGTTTAATTATAT

GGAATGTTGCTGTTAAAGAATCTTCTAATGCTCATCACCACCATCATCAC

TAA

>27_blue_Q_20m

ATGAAATTATTA

CTGATATTAGGTAGTGTTATTGCACTTCCTACATTTGCTGCAGGTGGTGG

TGACCTTGATGCTAGTGATTACACTGGTGTTTCTTTTTGGTTAGTTACTG

CTGCTTTATTAGCATCTACTGTATTTTTCTTTGTTGAAAGAGATAGAGTT

TCTGCAAAATGGAAAACATCATTAACTGTATCTGGTCTTGTTACTGGTAT

TGCTTTCTGGCATTACATGTACATGAGAGGGGTATGGATTGAAACTGGTG

ATTCGCCAACTGTATTTAGGTACCTAGATTGGTTGCTAACAGTACCTTTA

CAGATGGTAGAATTTTACCTAATTCTAGCTGCTGTTACTGTTGTAGCTGG

TTCTTTGTTCTGGCAATTGCTTTTAGGTTCTTTAGTCATGTTGATTTTTG

GTTACATGGGTGAGGCTGGACTTATGGCTGCAATGCCTGCTTTTGTAATT

GGAATGCTGGCTTGGCTTTACATGATCTATGTACTTTACATGGGTGCTGG

TAAAGCTGCTGTTTCCTCAACAAGCGCGTCAGTTCAAACTGCATATAATT

CAATGCTATTGATTATTGTTGTTGGCTGGTGCATTTATCCCGCCGGCTAT

TTCACAGGTTACCTGATGGGTGACGGTGGATCAGCTCTTAACTTAAACCT

TATCTATAACCTTGCTGACTTTGTTAACAAGATTCTATTTGGTTTAATTA

TATGGAATGTTGCTGTTAAAGAATCTTCTAATGCTCATCACCACCATCAT

CACTAA

>28_green_L_20m

ATGAAATTATT

ACTGATATTAGGTAGTGTTATTGCACTTCCTACATTTGCTGCAGGTGGTG

GTGACCTTGATGCTAGTGATTACACTGGTGTTTCTTTTTGGTTAGTTACT

GCTGCTTTATTAGCATCTACTGTATTTTTCTTTGTTGAAAGAGATAGAGT

TTCTGCAAAATGGAAAACATCATTAACTGTATCTGGTCTTGTTACTGGTA

TTGCTTTCTGGCATTACATGTACATGAGAGGGGTATGGATTGAAACTGGT

GATTCGCCAACTGTATTTAGGTACCTTGATTGGTTAATTACTGTGCCATT

ACTAATGGTTGAACTTTATATTGTTCTTCTTGCAGTAACTAAAGTAAGCG

CAGGAGTTTTCCACAGACTTCTTGGTTTCTCAACACTAATGCTTGTTTTC

GGGTATTTAGGAGAATCAGGTGCAGTTGCTGCTATTAACTCAACTACAGG

TTTTGTTGGTGGTATGCTTTTCTGGGGTCTATGCCTTAGAGAACTATGGT

CAGGTGAAGCTGCTGAAGCTAACGCTGGATCAAAAAATGCTGCAGGTCAC

TATGCGTATGATAGTCTAAAGAAAATTGTAACATTTGGTTGGGCTATTTA

CCCGGCCGGCTATTTCACAGGTTACCTGATGGGTGACGGTGGATCAGCTC

TTAACTTAAACCTTATCTATAACCTTGCTGACTTTGTTAACAAGATTCTA

TTTGGTTTAATTATATGGAATGTTGCTGTTAAAGAATCTTCTAATGCTCA

TCACCACCATCATCACTAA

>29_green_M_40m

ATGAAATTATT

ACTGATATTAGGTAGTGTTATTGCACTTCCTACATTTGCTGCAGGTGGTG

GTGACCTTGATGCTAGTGATTACACTGGTGTTTCTTTTTGGTTAGTTACT

GCTGCTTTATTAGCATCTACTGTATTTTTCTTTGTTGAAAGAGATAGAGT

TTCTGCAAAATGGAAAACATCATTAACTGTATCTGGTCTTGTTACTGGTA

TTGCTTTCTGGCATTACATGTACATGAGAGGGGTATGGATTGAAACTGGT

GATTCGCCAACTGTATTTAGGTACCTTGACTGGATCTTAACAGTCCCGTT

GATGTGTGTTGAGTTTTATCTATTAACGAAAACTGGTTTGAGTAAAATGG

TTTCAGCTTCTGTTGTTATGCTTGTTACAGGTTATCTTGGCGAAGCATCA

GTTTGTTCAGTTTTTGGTACTCATAGCCCTGCAATATGGGGATTGATTAG

CGGTTTGGCGTATTTTTATATCGTCAATGAAGTGTACCAAGGCGATGTAG

CAAAAGCAGCAAAAAAAGCTGGTAAAAATATTCAAAATGCCAACTCACTT

CTGTTAAAGTTCGTTGTAATTGGGTGGACCATTTATCCTGCCGGCTATTT

CACAGGTTACCTGATGGGTGACGGTGGATCAGCTCTTAACTTAAACCTTA

TCTATAACCTTGCTGACTTTGTTAACAAGATTCTATTTGGTTTAATTATA

TGGAATGTTGCTGTTAAAGAATCTTCTAATGCTCATCACCACCATCATCA

CTAA

>30_blue_Q_40m

ATGAAATTAT

TACTGATATTAGGTAGTGTTATTGCACTTCCTACATTTGCTGCAGGTGGT

GGTGACCTTGATGCTAGTGATTACACTGGTGTTTCTTTTTGGTTAGTTAC

TGCTGCTTTATTAGCATCTACTGTATTTTTCTTTGTTGAAAGAGATAGAG

TTTCTGCAAAATGGAAAACATCATTAACTGTATCTGGTCTTGTTACTGGT

ATTGCTTTCTGGCATTACATGTACATGAGAGGGGTATGGATTGAAACTGG

TGATTCGCCAACTGTATTTAGGTACCTGGACTGGATTTTAACTGTACCGT

TACAAATGATCGAATTCTACTTAATACTAGCAGCTGTAACAACTGTTAGT

GCAGGTGTATTCAATAGACTGCTTGTAGGTACACTAGTCATGTTGATTGG

TGGTTACTTAGGTGAAGCTGGAGTGATCAATGCGATGCTCGGATTCATCA

TCGGGATGGCAGGCTGGATATACATCCTGTATGAAATTTTTGCTGGTGAA

GCAGGACAAAAAAGTACGTCTTGTGGAAGCGTGGGTGCACAAATGGCATT

CAATGCATGTCGTATGATCGTATTAGTCGGGTGGTGTATCTATCCAGCCG

GCTATTTCACAGGTTACCTGATGGGTGACGGTGGATCAGCTCTTAACTTA

AACCTTATCTATAACCTTGCTGACTTTGTTAACAAGATTCTATTTGGTTT

AATTATATGGAATGTTGCTGTTAAAGAATCTTCTAATGCTCATCACCACC

ATCATCACTAA

>31_green_M_40m

ATGAAATTAT

TACTGATATTAGGTAGTGTTATTGCACTTCCTACATTTGCTGCAGGTGGT

GGTGACCTTGATGCTAGTGATTACACTGGTGTTTCTTTTTGGTTAGTTAC

TGCTGCTTTATTAGCATCTACTGTATTTTTCTTTGTTGAAAGAGATAGAG

TTTCTGCAAAATGGAAAACATCATTAACTGTATCTGGTCTTGTTACTGGT

ATTGCTTTCTGGCATTACATGTACATGAGAGGGGTATGGATTGAAACTGG

TGATTCGCCAACTGTATTTAGGTACCTTGACTGGATCTTAACAGTCCCGT

TGATGTGTGTTGAGTTTTATCTATTAACAAAAACTGGTTTGAGTAAAATG

GTTTCAGCTTCTGTTGTTATGCTTGTTACAGGTTATCTTGGCGAAGCATC

AGTTTGTTCAGTTTTTGGTACTCATAGCCCTGCAATATGGGGATTGATTA

GCGGTTTGGCGTATTTTTATATCGTCAATGAAGTGTACCAAGGCGATGTA

GCAAAAGCAGCAAAAAAAGCTGGTAAAAATATTCAAAATGCCAACTCACT

TCTGTTAAAGTTCGTTGTAATCGGTTGGAGCATATACCCTGCCGGCTATT

TCACAGGTTACCTGATGGGTGACGGTGGATCAGCTCTTAACTTAAACCTT

ATCTATAACCTTGCTGACTTTGTTAACAAGATTCTATTTGGTTTAATTAT

ATGGAATGTTGCTGTTAAAGAATCTTCTAATGCTCATCACCACCATCATC

ACTAA

>32_blue_Q_60m

ATGAAATTAT

TACTGATATTAGGTAGTGTTATTGCACTTCCTACATTTGCTGCAGGTGGT

GGTGACCTTGATGCTAGTGATTACACTGGTGTTTCTTTTTGGTTAGTTAC

TGCTGCTTTATTAGCATCTACTGTATTTTTCTTTGTTGAAAGAGATAGAG

TTTCTGCAAAATGGAAAACATCATTAACTGTATCTGGTCTTGTTACTGGT

ATTGCTTTCTGGCATTACATGTACATGAGAGGGGTATGGATTGAAACTGG

TGATTCGCCAACTGTATTTAGGTACCTTGATTGGTTGTTAACGGTACCTC

TTCAAATGGTTGAATTCTATTTGATTTTAGCAGCTGTTACAGCTGTTGCT

GGATCTTTATTCTGGCAACTTTTACTGGGTTCACTAGTAATGCTAATTTT

TGGTTTCATGGGAGAAGCTGGAATTATGGCGGCTATGCCTGCATTTGTTA

TCGGAATGGCAGCGTGGTTATACATGATTTATGTTCTCTACATGGGCGCA

GGTAAATCTGTAGTATCTACTACTAGTGCTTCGGTTCAAACAGCATACAA

CTCATTGCTATTAATCATTGTAGTTGGCTGGTGCATTTACCCTGCCGGCT

ATTTCACAGGTTACCTGATGGGTGACGGTGGATCAGCTCTTAACTTAAAC

CTTATCTATAACCTTGCTGACTTTGTTAACAAGATTCTATTTGGTTTAAT

TATATGGAATGTTGCTGTTAAAGAATCTTCTAATGCTCATCACCACCATC

ATCACTAA

>33_green_M_60m

ATGAAATTAT

TACTGATATTAGGTAGTGTTATTGCACTTCCTACATTTGCTGCAGGTGGT

GGTGACCTTGATGCTAGTGATTACACTGGTGTTTCTTTTTGGTTAGTTAC

TGCTGCTTTATTAGCATCTACTGTATTTTTCTTTGTTGAAAGAGATAGAG

TTTCTGCAAAATGGAAAACATCATTAACTGTATCTGGTCTTGTTACTGGT

ATTGCTTTCTGGCATTACATGTACATGAGAGGGGTATGGATTGAAACTGG

TGATTCGCCAACTGTATTTAGGTACCTGGACTGGATCTTAACAGTCCCGT

TGATGTGTGTTGAGTTTTATCTATTAACAAAAACTGGTTTGAGTAAAATG

GTTTCAGCTTCTGTTGTTATGCTTGTTACAGGTTATCTTGGCGAAGCATC

AGTTTGTTCAGTTTTTGGTACTCATAGCCCTGCAATATGGGGATTGATTA

GCGGTTTGGCGTATTTTTATATCGTCAATGAAGTGTACCAAGGCGATGTA

GCAAAAGCAGCAAAAAAAGCTGGTAAAAATATTCAAAATGCCAATTCGCT

TCTGTTAAAGTTCGTTGTAATTGGTTGGGGAATATACCCTGCCGGCTATT

TCACAGGTTACCTGATGGGTGACGGTGGATCAGCTCTTAACTTAAACCTT

ATCTATAACCTTGCTGACTTTGTTAACAAGATTCTATTTGGTTTAATTAT

ATGGAATGTTGCTGTTAAAGAATCTTCTAATGCTCATCACCACCATCATC

ACTAA

>34_blue_Q_60m

ATGAAATTAT

TACTGATATTAGGTAGTGTTATTGCACTTCCTACATTTGCTGCAGGTGGT

GGTGACCTTGATGCTAGTGATTACACTGGTGTTTCTTTTTGGTTAGTTAC

TGCTGCTTTATTAGCATCTACTGTATTTTTCTTTGTTGAAAGAGATAGAG

TTTCTGCAAAATGGAAAACATCATTAACTGTATCTGGTCTTGTTACTGGT

ATTGCTTTCTGGCATTACATGTACATGAGAGGGGTATGGATTGAAACTGG

TGATTCGCCAACTGTATTTAGGTACCTCGACTGGCTATTAACCGTGCCAC

TACAAATGATTGAATTCTATCTGATCTTAGCTGCAGTAACTGCAGTAAGC

GCAGGCGTGTTCTATAGATTATTAGTTGGTACATTAGTTATGTTGGTTGC

AGGATATGCTGGTGAGGCTGGCATTATCAATGCAGCACTAGGATTTGTTA

TTGGTATGGCTGGATGGATATACATTCTATATGAAATCTTTTCCGGTGAA

GCTGGGCAAGTTAGTAAAACTGTCAGTGCATCAGTAGCATCAGCATTCGC

AGCTTGTCGTATGGTTGTACTCGTTGGTTGGTCAATATATCCAGCCGGCT

ATTTCACAGGTTACCTGATGGGTGACGGTGGATCAGCTCTTAACTTAAAC

CTTATCTATAACCTTGCTGACTTTGTTAACAAGATTCTATTTGGGTTAAT

TATATGGAATGTTGCTGTTAAAGAATCTTCTAATGCTCATCACCACCATC

ATCACTAA

>35_blue_Q_60m

ATGAAATTAT

TACTGATATTAGGTAGTGTTATTGCACTTCCTACATTTGCTGCAGGTGGT

GGTGACCTTGATGCTAGTGATTACACTGGTGTTTCTTTTTGGTTAGTTAC

TGCTGCTTTATTAGCATCTACTGTATTTTTCTTTGTTGAAAGAGATAGAG

TTTCTGCAAAATGGAAAACATCATTAACTGTATCTGGTCTTGTTACTGGT

ATTGCTTTCTGGCATTACATGTACATGAGAGGGGTATGGATTGAAACTGG

TGATTCGCCAACTGTATTTAGGTACCTAGATTGGTTAATCACTGTGCCTT

TACAGATGGTAGAATTCTATTTAATCCTAGCAGCTATCGGTAAAGCAAAC

TCTGGAATGTTCTGGAGATTGTTAATCGGTTCATTAGTAATGCTTATCGG

TGGTTACTTAGGTGAAGCAGGATATATAAATGCTACACTAGGTTTCATCA

TCGGAATGGCAGGTTGGGTATACATTCTTTATGAAGTATTCTCAGGTGAA

GCTGGAAAAGCTGCAGCGAAGAGTGGTAACAAAGCTCTTGTAACTGCATT

TGGAGCAATGAGAATGATTGTTACAGTAGGTTGGAGCATTTATCCCGCCG

GCTATTTCACAGGTTACCTGATGGGTGACGGTGGATCAGCTCTTAACTTA

AACCTTATCTATAACCTTGCTGACTTTGTTAACAAGATTCTATTTGGTTT

AATTATATGGAATGTTGCTGTTAAAGAATCTTCTAATGCTCATCACCACC

ATCATCACTAA

>36_green_Q_60m

ATGAAATTAT

TACTGATATTAGGTAGTGTTATTGCACTTCCTACATTTGCTGCAGGTGGT

GGTGACCTTGATGCTAGTGATTACACTGGTGTTTCTTTTTGGTTAGTTAC

TGCTGCTTTATTAGCATCTACTGTATTTTTCTTTGTTGAAAGAGATAGAG

TTTCTGCAAAATGGAAAACATCATTAACTGTATCTGGTCTTGTTACTGGT

ATTGCTTTCTGGCATTACATGTACATGAGAGGGGTATGGATTGAAACTGG

TGATTCGCCAACTGTATTTAGGTACCTTGATTGGCTATTAACCGTGCCAC

TACAAATGATTGAATTCTATCTGATCTTAGCTGCAGTAACTGCAGTAAGC

GCAGGAGTGTTCTATAGATTGTTAGTTGGTACATTAGTTATGTTGGTTGC

AGGATATGCTGGTGAGGCTGGCATTATCAATGCAGCACTCGGATTTGTTA

TTGGTATGGCTGGATGGATATACATTCTATATGAAATCTTTTCCGGTGAA

GCTGGGCAAGTTAGTAAAACTGTCAGCGCATCAGTAGCATCAGCATTCGC

AGCTTATCGTATGGTTGTACTTGTTGGGTGGACTATATATCCTGCCGGCT

ATTTCACAGGTTACCTGATGGGTGACGGTGGATCAGCTCTTAACTTAAAC

CTTATCTATAACCTTGCTGACTTTGTTAACAAGATTCTATTTGGTTTAAT

TATATGGAATGTTGCTGTTAAAGAATCTTCTAATGCTCATCACCACCATC

ATCACTAA

>37_green_Q_60m

ATGAAATTATTACTGATATTAGGTAGTGT

TATTGCACTTCCTACATTTGCTGCAGGTGGTGGTGACCTTGATGCTAGTG

ATTACACTGGTGTTTCTTTTTGGTTAGTTACTGCTGCTTTATTAGCATCT

ACTGTATTTTTCTTTGTTGAAAGAGATAGAGTTTCTGCAAAATGGAAAAC

ATCATTAACTGTATCTGGTCTTGTTACTGGTATTGCTTTCTGGCATTACA

TGTACATGAGAGGGGTATGGATTGAAACTGGTGATTCGCCAACTGTATTT

AGGTACCTCGATTGGTTAATTACTGTACCTTTACAGCTAATAGAATTTTA

TCTAATTCTTTCAGCAGTAAGAAAAGTTGACAGCGGCATTTTCTGGAGAA

TCTTTATCGGTTCTTTAGTGATGTTAGTTGGAGGATATCTTGGAGAAGCA

GGATTCATCAACTCTATGCTTGGTTTCATTATCGGTATGGCAGGATGGAT

TTACATTCTTTATGAAATATTCTCAGGTGAAGCTGGGAAAGCAGTTTCTA

AATCTGGAAACAAAGCACTTGTTACAGCATTTGGAGCTTTAAGAATGATC

GTTACTGTAGGGTGGGCTATTTACCCTGCCGGCTATTTCACAGGTTACCT

GATGGGTGACGGTGGATCAGCTCTTAACTTAAACCTTATCTATAACCTTG

CTGACTTTGTTAACAAGATTCTATTTGGTTTAATTATATGGAATGTTGCT

GTTAAAGAATCTTCTAATGCTCATCACCACCATCATCACTAA

>38_blue_Q_60m

ATGAAATTATT

ACTGATATTAGGTAGTGTTATTGCACTTCCTACATTTGCTGCAGGTGGTG

GTGACCTTGATGCTAGTGATTACACTGGTGTTTCTTTTTGGTTAGTTACT

GCTGCTTTATTAGCATCTACTGTATTTTTCTTTGTTGAAAGAGATAGAGT

TTCTGCAAAATGGAAAACATCATTAACTGTATCTGGTCTTGTTACTGGTA

TTGCTTTCTGGCATTACATGTACATGAGAGGGGTATGGATTGAAACTGGT

GATTCGCCAACTGTATTTAGGTACCTGGACTGGTTTATTACGGTTCCTCT

TCAAATTGTTGAGTTTTATCTGATATTAGCAGCAGTAACTATAGTAAGTC

ATAAACTATTCTGGAAACTTCTTGTATCTAGTTTAGTTATGTTAATTGGT

GGCTACTGTGGTGAGACTTGTGCTGGTTGGCAGACTCAAGGATTTGTAAT

CGGAATGATAGGTTGGATTGGCGTCTTATACCTAATTTTCATGGGTGATG

CAGCGAAAGCTAATCAGGCAAGTGGTAATAAGTCTAGTCAGTTTGCATTT

AAAGTAATGAGATTGATTGTTTTAGTCGGCTGGTCAATATACCCCGCCGG

CTATTTCACAGGTTACCTGATGGGTGACGGTGGATCAGCTCTTAACTTAA

ACCTTATCTATAACCTTGCTGACTTTGTTAACAAGATTCTATTTGGGTTA

ATTATATGGAATGTTGCTGTTAAAGAATCTTCTAATGCTCATCACCACCA

TCATCACTAA

>39_blue_Q_60m

ATGAAATTATTA

CTGATATTAGGTAGTGTTATTGCACTTCCTACATTTGCTGCAGGTGGTGG

TGACCTTGATGCTAGTGATTACACTGGTGTTTCTTTTTGGTTAGTTACTG

CTGCTTTATTAGCATCTACTGTATTTTTCTTTGTTGAAAGAGATAGAGTT

TCTGCAAAATGGAAAACATCATTAACTGTATCTGGTCTTGTTACTGGTAT

TGCTTTCTGGCATTACATGTACATGAGAGGGGTATGGATTGAAACTGGTG

ATTCGCCAACTGTATTTAGGTACCTCGATTGGCTAATCACAGTACCACTG

CAAGTTGTTGAGTTCTACCTAATCCTCGCCGCTATCGGTGTGGGAACTTT

CGCCATGTTCAGAAACCTCATGGGAGCATCTATCGTCATGCTAGTGGCTG

GATTCTTCGGTGAGTCCGGAGCTATGGACAGTACAATCGATCTATCCGCT

GAGATCTGGTGGGTCATCGGAATGGCTGGATGGGGATACATCCTATACGA

GCTATGGTCCGGAGATGTCAAAGAGGCTTCAGAGACTGGTAGCCCAAGCG

TTCAGTACGCATTCAACTCAATGAGGATGATTGTAACTGTAGGGTGGTGC

ATATACCCTGCCGGCTATTTCACAGGTTACCTGATGGGTGACGGTGGATC

AGCTCTTAACTTAAACCTTATCTATAACCTTGCTGACTTTGTTAACAAGA

TTCTATTTGGTTTAATTATATGGAATGTTGCTGTTAAAGAATCTTCTAAT

GCTCATCACCACCATCATCACTAA

>40_blue_Q_80m

ATGAAATTA

TTACTGATATTAGGTAGTGTTATTGCACTTCCTACATTTGCTGCAGGTGG

TGGTGACCTTGATGCTAGTGATTACACTGGTGTTTCTTTTTGGTTAGTTA

CTGCTGCTTTATTAGCATCTACTGTATTTTTCTTTGTTGAAAGAGATAGA

GTTTCTGCAAAATGGAAAACATCATTAACTGTATCTGGTCTTGTTACTGG

TATTGCTTTCTGGCATTACATGTACATGAGAGGGGTATGGATTGAAACTG

GTGATTCGCCAACTGTATTTAGGTACCTGGATTGGTTGATCACAGTTCCG

CTGCAGATGGTTGAATTCTACTTCATCCTCGCTGCTGTTACTGCAGTCAG

TGCTGGCATTTTCTGGCGGCTACTGATTGGATCGCTCGTAATGTTGGTCG

CAGGTTACGCCGGTGAAGCCGGCCTAATAAACGCCTGGGCTGGCTTTATC

GTAGGTTTGGCAGGATGGGCTTACATCTTGTATGAAATCTTTGCTGGTGA

GGCGGGCAGAGCTTCTGCAGATAAAGCCCCAGCATCGGTGCAAAGTGCCT

TCGGAACGATGCGGTTGATCGTAACAGTCGGATGGACCATATACCCTGCC

GGCTATTTCACAGGTTACCTGATGGGTGACGGTGGATCAGCTCTTAACTT

AAACCTTATCTATAACCTTGCTGACTTTGTTAACAAGATTCTATTTGGTT

TAATTATATGGAATGTTGCTGTTAAAGAATCTTCTAATGCTCATCACCAC

CATCATCACTAA

>41_green_M_100m

ATGAAATTA

TTACTGATATTAGGTAGTGTTATTGCACTTCCTACATTTGCTGCAGGTGG

TGGTGACCTTGATGCTAGTGATTACACTGGTGTTTCTTTTTGGTTAGTTA

CTGCTGCTTTATTAGCATCTACTGTATTTTTCTTTGTTGAAAGAGATAGA

GTTTCTGCAAAATGGAAAACATCATTAACTGTATCTGGTCTTGTTACTGG

TATTGCTTTCTGGCATTACATGTACATGAGAGGGGTATGGATTGAAACTG

GTGATTCGCCAACTGTATTTAGGTACCTGGACTGGATCTTAACAGTCCCG

TTGATGTGTGTTGAGTTTTATCTATTAACAAACACTGGTTTGAGTAAAAT

GGTTTCAGCTTCTGTTGTTATGCTTGTTACAGGTTATCTTGGCGAAGCAT

CAGTTTGTTCAGTTTTTGGTACTCATAGCCCTGCAATATGGGGATTGATT

AGCGGTTTGGCGTATTTTTATATCGTCAATGAAGTGTACCAAGGCGATGT

AGCAAAAGCAGCAAAAAAAGCTGGTAAAAATATTCAAAATGCCAATTCGC

TTCTGTTAAAGTTCGTTGTAATTGGGTGGTCGATTTACCCCGCCGGCTAT

TTCACAGGTTACCTGATGGGTGACGGTGGATCAGCTCTTAACTTAAACCT

TATCTATAACCTTGCTGACTTTGTTAACAAGATTCTATTTGGTTTAATTA

TATGGAATGTTGCTGTTAAAGAATCTTCTAATGCTCATCACCACCATCAT

CACTAA

>42_blue_Q_100m

ATGAAATTAT

TACTGATATTAGGTAGTGTTATTGCACTTCCTACATTTGCTGCAGGTGGT

GGTGACCTTGATGCTAGTGATTACACTGGTGTTTCTTTTTGGTTAGTTAC

TGCTGCTTTATTAGCATCTACTGTATTTTTCTTTGTTGAAAGAGATAGAG

TTTCTGCAAAATGGAAAACATCATTAACTGTATCTGGTCTTGTTACTGGT

ATTGCTTTCTGGCATTACATGTACATGAGAGGGGTATGGATTGAAACTGG

TGATTCGCCAACTGTATTTAGGTACCTGGACTGGCTAATAACAGTCCCTC

TACAGGTGGTAGAGTTCTACCTAATCCTCGCCGCTATCGGTGTGGGAACT

TTCGTCATGTTCAGGAACCTCATGGTAGCATCCTTAGTCATGCTAGTAGC

AGGATTCTTCGGAGAGTCCGGAGCTATGGATGGGACTGCTCTCGAGTTCT

CAGCAGAAATCTGGTGGGTCATTGGAATGGCAGGATGGGGATACATCCTA

TACGAGCTATGGAAGGGTGACGTCAAGGAGGCATCTGCTTCTGGTAGCGA

GGGCGTCCAGTACGCCTTCACCTCGATGAGGTGGATTGTTACCGCCGGCT

GGGCGATATATCCGGCCGGCTATTTCACAGGTTACCTGATGGGTGACGGT

GGATCAGCTCTTAACTTAAACCTTATCTATAACCTTGCTGACTTTGTTAA

CAAGATTCTATTTGGTTTAATTATATGGAATGTTGCTGTTAAAGAATCTT

CTAATGCTCATCACCACCATCATCACTAA

>43_green_M_100m

ATGAAATTAT

TACTGATATTAGGTAGTGTTATTGCACTTCCTACATTTGCTGCAGGTGGT

GGTGACCTTGATGCTAGTGATTACACTGGTGTTTCTTTTTGGTTAGTTAC

TGCTGCTTTATTAGCATCTACTGTATTTTTCTTTGTTGAAAGAGATAGAG

TTTCTGCAAAATGGAAAACATCATTAACTGTATCTGGTCTTGTTACTGGT

ATTGCTTTCTGGCATTACATGTACATGAGAGGGGTATGGATTGAAACTGG

TGATTCGCCAACTGTATTTAGGTACCTGGATTGGATCTTAACAGTCCCGT

TGATGTGTGTTGAGTTTTATCTATTAACAAAAACTGGTTTGAGTAAAATG

GTTTCAGCTTCTGTTGTTATGCTTGTTACAGGTTATCTTGGCGAAGCATC

AGTTTGTTCAGTTTTTGGTACTCATAGCCCTGCAATATGGGGATTGATTA

GCGGTTTGGCGTATTTTTATATCGTCAATGAAGTGTACCAAGGCGATGTA

GCAAAAGCAGCAAAAAAAGCTGGTAAAAATATTCAAAATGCCAACTCGCT

TCTGTTAAAGTTCGTTGTAATTGGGTGGTCCATCTACCCTGCCGGCTATT

TCACAGGTTACCTGATGGGTGACGGTGGATCAGCTCTTAACTTAAACCTT

ATCTATAACCTTGCTGACTTTGTTAACAAGATTCTATTTGGTTTAATTAT

ATGGAATGTTGCTGTTAAAGAATCTTCTAATGCTCATCACCACCATCATC

ACTAA

>44_green_Q_100m

ATGAAATTAT

TACTGATATTAGGTAGTGTTATTGCACTTCCTACATTTGCTGCAGGTGGT

GGTGACCTTGATGCTAGTGATTACACTGGTGTTTCTTTTTGGTTAGTTAC

TGCTGCTTTATTAGCATCTACTGTATTTTTCTTTGTTGAAAGAGATAGAG

TTTCTGCAAAATGGAAAACATCATTAACTGTATCTGGTCTTGTTACTGGT

ATTGCTTTCTGGCATTACATGTACATGAGAGGGGTATGGATTGAAACTGG

TGATTCGCCAACTGTATTTAGGTACCTGGACTGGTTAATCACAGTTCCAC

TTCAAATCATTGAGTTTTACTTGATTGTTGCAGCTGTTACAGCGGTAAGC

GCAGGCATATTCTGGCGTTTACTTATCGCATCAATAGTAATGCTTGTTGG

TGGTTACCTTGGTGAAACCGGACTATGGGCACCTTCAGTAGGTTTTGCTG

TCGGAATGATTGCTTGGGTATACATCATCTATGAAATATTTCTTGGTGAA

ACTGCCGCAGCAAATGCCTCTAGTGGAAACTCTGCAAGTCAAAGCGCATT

TAATACGATTAAATGGATTGTAACTGTAGGGTGGGTAATATACCCTGCCG

GCTATTTCACAGGTTACCTGATGGGTGACGGTGGATCAGCTCTTAACTTA

AACCTTATCTATAACCTTGCTGACTTTGTTAACAAGATTCTATTTGGTTT

AATTATATGGAATGTTGCTGTTAAAGAATCTTCTAATGCTCATCACCACC

ATCATCACTAA

>45_blue_Q_100m

ATGAAATTAT

TACTGATATTAGGTAGTGTTATTGCACTTCCTACATTTGCTGCAGGTGGT

GGTGACCTTGATGCTAGTGATTACACTGGTGTTTCTTTTTGGTTAGTTAC

TGCTGCTTTATTAGCATCTACTGTATTTTTCTTTGTTGAAAGAGATAGAG

TTTCTGCAAAATGGAAAACATCATTAACTGTATCTGGTCTTGTTACTGGT

ATTGCTTTCTGGCATTACATGTACATGAGAGGGGTATGGATTGAAACTGG

TGATTCGCCAACTGTATTTAGGTACCTTGATTGGTTAATCACAGTACCAC

TACAAGTCGTAGAATTCTACCTAATTCTAGCGGCAATCGGAGTGGGTACT

GCAATTATGTTCCAGAGACTACTTGGGGCATCTATTGTTATGCTGGTTGC

AGGATACTTCGGTGAATCCGGAGCTATGGACAGTACTCTGGAACTATCAC

CAACAGTCTGGTGGATAATCGGAATGGCCGGATGGGCATGGATTCTCTAC

GAACTATGGGCTGGAGAAGTCGGTGCAGCTGCAGAGAGCGGAAGCGCAGG

AGTCCAATATGGATTCAATGCAATGAAGTTGATTGTAACATTTGGCTGGA

CTATCTACCCGGCCGGCTATTTCACAGGTTACCTGATGGGTGACGGTGGA

TCAGCTCTTAACTTAAACCTTATCTATAACCTTGCTGACTTTGTTAACAA

GATTCTATTTGGTTTAATTATATGGAATGTTGCTGTTAAAGAATCTTCTA

ATGCTCATCACCACCATCATCACTAA
